# Anti-Inflammatory and Anti-Quorum Sensing Effect of *Camellia sinensis* Callus Lysate for Treatment of Acne

**DOI:** 10.3390/cimb45050255

**Published:** 2023-05-04

**Authors:** Mariona Cañellas-Santos, Elisabet Rosell-Vives, Laia Montell, Ainhoa Bilbao, Felipe Goñi-de-Cerio, Francisco Fernandez-Campos

**Affiliations:** 1 Laboratory Reig Jofre, Avda del Flors s/n, 08970 Sant Joan Despí, Barcelona, Spain; 2 GAIKER Technology Centre, Basque Research and Technology Alliance (BRTA), Parque Tecnológico de Bizkaia, Edif. 202, 48170 Zamudio, Spain

**Keywords:** *Camellia sinensis*, *Cutibacterium acnes*, keratinocytes, biofilm, quorum sensing, anti-inflammatory cytokines, callus lysate, acne

## Abstract

*Cutibacterium acnes* (*C. acnes*) is involved in the pathogenesis of acne by inducing inflammation and biofilm formation, along with other virulence factors. A *Camellia sinensis* (*C. sinensis*) callus lysate is proposed to reduce these effects. The aim of the present work is to study the anti-inflammatory properties of a callus extract from *C. sinensis* on *C. acnes*-stimulated human keratinocytes and the quorum-quenching activities. Keratinocytes were stimulated with thermo-inactivated pathogenic *C. acnes* and were treated with the herbal lysate (0.25% *w*/*w*) to evaluate its anti-inflammatory effect. *C. acnes* biofilm was developed in vitro and treated with 2.5 and 5% *w*/*w* of the lysate to evaluate quorum sensing and the lipase activity. The results showed that the lysate was able to reduce the production of interleukin-6 (IL-6), interleukin-8 (IL-8), tumor necrosis factor-α (TNF-α), and C-X-C motif chemokine ligand 1 (CXCL1), and decrease the nuclear translocation of nuclear factor kappa light chain enhancer of activated B cells (NF-κB). The lysate did not show bactericidal activity but showed diminished biofilm formation, the lipase activity, and the production of autoinducer 2 (AI-2), a member of a family of signaling molecules used in quorum sensing. Therefore, the proposed callus lysate could have the potential to reduce acne-related symptoms without the eradication of *C. acnes,* which is part of the natural skin microbiome.

## 1. Introduction

Acne vulgaris is a chronic, inflammatory skin condition that involves the pilosebaceous follicles and is influenced by a variety of factors including genetics, the androgen-stimulation of sebaceous glands with abnormal keratinization, colonization with *C. acnes*, and pathological immune response to inflammation [1,2]. On the skin, *C. acnes* will proliferate until it has reached a high population density and will thereafter form a biofilm through the excretion of the extracellular matrix, which will decrease its sensitivity to xenobiotics [3,4]. This phenomenon is induced via quorum sensing, a system that allows bacteria-to-bacteria communication and which is a key factor in the development of virulence factors, such as biofilm formation and antibiotic resistance. There are several molecules implicated in the quorum-sensing pathway, such as auto-inducer 2 (AI-2), which is well-characterized and widespread among several bacterial species [5], including *C. acnes* [6]. Some of these pathways can be modified to prevent bacterial overgrowth and virulence induction, without using wide-spectrum antibiotics that kill pathogenic as well as commensal bacteria.

Different therapeutic approaches are currently available to treat acne. Topical retinoids (such as tretinoin, adapalene, etc.) have become the gold-standard treatment, together with oral retinoids in severe cases [7,8,9]. Depending on acne severity, topical retinoids can be used combined with topical benzoyl peroxide (BP) and topical or oral antibiotics [10,11]. Both retinoids and BP are highly effective against acne, but they induce skin irritation and photosensitizing. In addition, antimicrobial resistance is a very important disadvantage of these products [7].

Therefore, since conventional therapies have not demonstrated the desirable effectiveness and possess remarkable side effects, there is a growing interest in the use of herbal medicines for the management of acne vulgaris.

The antibacterial and anti-inflammatory potential of natural, plant-derived compounds has been reported in many studies. In vitro and in vivo studies have revealed that essential oils mixture, oleoresin, flavonoids, alkaloids, phenol and phenolic compounds, tannin, xanthone and xanthone derivatives, diterpene acid, phenylpropanoid glycosides, acteoside, and the bisnaphthquione derivatives are effective in the treatment of acne due to their antimicrobial and anti-inflammatory activities [12,13]. Many plant-derived procedures involve the use of different plant extracts, for example, *Myrtus* extracts [14] and *Helichrysum Odoratissimum* [15,16]. *C. sinensis* or green tea extracts have been associated with health benefits since antiquity [17]. The principal mediators of these biological effects are polyphenol catechins and terpenes, which promote a reduction of the expression of inflammatory associated genes, and an increase of the expression of antioxidative genes and antimicrobial activity [18,19,20,21,22,23]. 

Treatment with herbal extracts is an interesting approach, alone or as a coadjuvant, for mild-to-moderate acne; however, there are some drawbacks, for instance, batch-to-batch variability in the chemical concentrations of the active compounds, sometimes related with the climate, the available nutrients, etc.; the necessity of bast crops to support the increasing demand, which can lead to deforestation, the presence of plagues, and the use of pesticides; and the high carbon print resulting from being transported from the producing countries to where the products are manufactured. All these factors are important in light of the global challenges related to climate change and the resources crisis. Alternative biotechnological solutions, such as callus in vitro cultures, try to overcome these issues from a sustainable point of view.

The methodology for initiating the in vitro cultures of plant cells, tissues, and organs is nowadays well-established [24]. The cultures can be initiated from parts of the whole plant, or from seeds which are germinated aseptically. A prerequisite for introducing any plant material into an in vitro culture is surface sterilization in order to eliminate adhering micro-organisms. Suitable explants are then inoculated on a semi-solid medium containing adequate amounts of nutrients and, most importantly, plant growth regulators, and cultivated under controlled environmental conditions. Depending on the starting material and the nutrient medium, different types of cultures can be established which are classified as cell cultures, tissue cultures (callus or differentiated tissues), or organ cultures. The formation of callus (dedifferentiated cell masses) can be induced, and upon transfer to a liquid medium, clumps of callus might disintegrate into small aggregates and single cells, whereby cell suspension cultures are obtained. As callus are typically quite heterogeneous in terms of the biochemical properties of the cells, suspension cultures should be started from small callus aggregates, so that homogeneous cell lines, desirably with a fast growth, can evolve. Cell suspension cultures are a potential source for the production of high-value plant secondary metabolites, and, during the past decades, cultures from many medicinal plants have been established [24]. Therefore, the large number of studies on the in vitro production of bioactive plant compounds published in the past few years indicates the unabated importance of relevant research. Factors such as culture techniques or the impact of elicitation on gene expression appear to be of the utmost importance, and, for a number of medicinal plants, cell or organ cultures producing higher amounts of relevant metabolites than the respective plant have been reported. Furthermore, the biotechnological manufacturing of such compounds offers several advantages such as predictable, stable, and year-round sustainable production, scalability, and easier extraction and purification. Plant cell and tissue cultures represent one possible alternative to the extraction of phytochemicals from plant material [24].

In addition, in vitro cultures employing plant tissue culture technology is advantageous over intact plants for producing secondary metabolites without destroying the natural habitat. This is because the rate of cell growth and biosynthesis in cultures initiated from a small amount of plant material is quite high, considering the short period of time necessary for its production. Manipulating culture conditions, phytohormones are a valuable tool for increasing the level of bioactive metabolites. This contrasts with the large number of in vivo plants used to obtain a small quantity of the drug [25]. Tissue cultures are able to grow in vitro under controlled conditions in bioreactors. From the productive point of view, their culture can follow general good manufacturing practices (GMP), thus making it possible to obtain a reproducible product using a method that is easy to scale up [26,27]. There is a huge number of secondary metabolites of therapeutic interest, and they can be obtained from in vitro plant cell cultures. They are produced as a defense response in reaction to different stressors or elicitors (UV light, heat, cold, chemicals, etc.). The appropriate in vitro conditions and elicitors can be used to enrich the culture with the compounds of interest. Thus, alternative biotechnological plant product approaches, such as callus in vitro cultures, have recently emerged. 

In this sense, a *C. sinensis* callus lysate was developed to produce an anti-inflammatory response in keratinocytes and activity against bacterial growth. Considering that *C. acnes* is a commensal micro-organism, the aim of the treatment is not to kill the bacteria, but to limit its overgrowth and rebalance the skin microbiome, as an alternative approach to managing the disease. This could be achieved by blocking the quorum-sensing bacterial communication, as bacteria communicate with each other by means of quoro-hormones (such as AI-2). 

The study reported in this article describes the anti-inflammatory properties of this *C. sinensis* callus lysate on *C. acnes*-stimulated keratinocytes and the quorum-quenching activities, which limit biofilm formation and the expression of virulence factors.

## 2. Materials and Methods

### 2.1. Materials 

*C. sinensis* callus cell freeze-dried lysate from Reig Jofre (Barcelona, Spain) was used for the experimental procedures. For the chemical characterization of the herbal lysate, the following reagents were used: dichloromethane, sulfuric acid, acetonitrile (ACN), formic acid, and sodium carbonate obtained from Panreac (Barcelona, Spain). β-caryophyllene, green tea catechin mix, and epicatechin (Sigma-Aldrich, Sant Louis, MO, USA) were used as standard for terpene and catechin quantification, respectively. 

For in vitro cell culture experiments, the following reagents were used: HaCaT cell line (RRID:CVCL_0038, aneuploid immortal keratinocyte cell line from adult human skin, derived from a 62-year-old male) were kindly gifted by Department of Biochemistry and Molecular Biomedicine of the Faculty of Biology of the Universitat de Barcelona. DMEM high glucose, heat-inactivated fetal bovine serum, penicillin/streptomycin, Trypsin-EDTA 0.25%, nuclease-free Water, trypan blue, and phosphate-buffered saline (PBS) pH 7.4 were obtained from Fisher Scientific (Hampton, VA, USA). The primers (see Supplementary Material) for real-time polymerase chain reaction (RT-PCR) Hs00174103_m1, Hs00174128_m1, Hs00174131_m1, Hs00236937_m1, Hs02786624_g1, and bovine serum albumin (BSA) Fraction V, Immunoglobulin G (IgG) Free, DAPI solution 1 mg / mL, and 16% paraformaldehyde (PFA) (*w*/*v*) methanol-free were obtained from ThermoFisher (Waltham, MA, USA). Antibodies anti-TLR2, anti-TLR4, PE Mouse IgG1, and κ Isotype Control were purchased from Becton Dickenson (Franklin Lakes, NJ, USA). Cell Counting Kit-8, Triton™ X-100, and Tween-20 were acquired from Sigma-Aldrich (Sant Louis, MO, USA). Other reagents employed were *C. acnes* (TISELAB, Cornella de Llobregat, Spain), the RNeasy Mini Kit (WERFEN, LLiça d’Amunt, Spain), iScript RT-PCR Supermix 100 (Bio-Rad, Hercules, CA, USA), and Anti-NF-κB p65 Antibody (F-6) Alexa Fluor^®^ 488 (Santa Cruz Biotechnology, Inc., Dallas, TX, USA).

For antibacterial studies, the following materials were employed. *Vibrio harveyi* (*V. harveyi*) ATCC^®^ BAA-1117 strain BB170 and *V. harveyi* ATCC^®^ BAA-1119 strain BB152 (LCG standards, Barcelona, Spain) were used for quorum-sensing studies. Brucella blood agar with hemin and vitamin K1 (BD life sciences, Madrid, Spain), reinforced clostridial medium (RCM), and tryptone and soya broth (TS) (ThermoFisher, Waltham, MA, USA) were used as growth medium for the bacterium. Anaerobic bacteria were cultured in the AnaeroGen system (ThermoFisher, Waltham, MA, USA), and 4-Methylumbelliferyl oleate (Sigma-Aldrich, Sant Louis, MO, USA) and the filmtracer LIVE/DEAD biofilm viability kit (ThermoFisher, Waltham, MA, USA) were used for fluorescence-based experiments.

### 2.2. Methods

#### 2.2.1. Total Terpene Quantification 

For total terpene quantification, the method described by Ghorai et al. [28] using β-caryophyllene as analytical standard was used. Briefly, freeze-dried lysate was diluted with water and sonicated in ice to prevent compound degradation. Then, samples were centrifuged at 1000× *g* for 15 min and the supernatant underwent liquid–liquid extraction with the same volume of dichloromethane. After three additional washing steps, the organic phase was mixed with concentrated sulfuric acid (5:2, *v*/*v*) and left to rest for 24 h before a spectrophotometric reading (wavelength 538 nm) was taken of the sulfuric phase. β-caryophyllene was used to generate the calibration curve (from 1500 to 15,000 µg). Measurements were performed in quintuplicate. This standard is for identification, not for quantification; then, the retention time was determined but the area of the peaks was not measured.

#### 2.2.2. Catechin Quantification by Liquid Chromatography-Mass Spectrometry (LC-MS)

A high-performance liquid chromatography-tandem mass spectrometry (HPLC-MS/MS) (Waters 2795 XE Separations Module and Micromass Quattro Ultima triple-stage quadrupole MS, Milford, MA, USA) was used to quantify the catechin profile of the herbal lysate. A mobile phase was composed of A: water (0.1% *v*/*v* formic acid) and B: ACN flowed through a C18 column (250 × 4.6 mm, 5 µm, at 30°C) under gradient elution at 1 mL/min (0–5 min: 100% of A; 8–30 min: 88% of A; 33–53 min: 83% of A). The injection volume was 20 µL, which correspond to 2.75 mg of dry lysate. A postcolumn split was performed before entry to the mass spectrometer, which was carried out using negative electrospray detection mode, a desolvation temperature of 350 °C, and a desolvation gas flow of 810 L/h. The green tea catechin standard mix contained caffeine, (+)-catechin, (−)-catechin 3-gallate, (−)-epicatechin, (−)-epicatechin-3-gallate, (−)-epigallocatechin 3-gallate, (−)-gallocatechin, and (−)-gallocatechin 3-gallate, and it was used to identify the corresponding retention time of each compound. Measurements were performed in quintuplicate.

#### 2.2.3. HaCaT Cells and *C. acnes* Stimuli 

The transformed immortal keratinocyte HaCaT cell line was used to evaluate the anti-inflammatory efficacy of the callus lysate. After thawing, the cell line was cultured for a minimum of 2 weeks before use. Cells were passaged before reaching 80% confluence. Cells were grown in a monolayer on a solid support at 37 °C in a humified atmosphere with 5% CO_2_ and maintained in DMEM media supplemented with 10% heat-inactivated fetal bovine serum and 1% penicillin/streptomycin.

For in vitro cell culture experiments, *C. acnes* ATCC 6919 (which corresponds to phylotype IA_1_) [29,30,31] was employed as stimuli. One freeze-dried bacteria pellet (equivalent to 2.4 × 10^7^ colony forming units (CFU)/pellet) was resuspended in 0.5 mL of sterile double-distilled water and heated at 80 °C for 20 min for the inactivation of the bacteria. 

#### 2.2.4. In Vitro Cytotoxicity Evaluation

HaCaT cells were seeded in three 96-well plates at 10,000 cells/well in 100 µL of media and cultured as described in Section 2.2.3. After 24 h, the cells were treated with 10 serial dilutions in cell media of the *C. sinensis* lysate (2.0 to 0.0%). Six replicates per concentration were carried out in each plate. Finally, Cell Counting Kit 8 (CCK8) was used to evaluate cell viability in the wells. Then, 20 µL of CCK8 reagent was added directly to every well plate cultured and incubated for 3.5 h. Absorbances at 450 nm were measured immediately using a Spectrophotometer Victor X3 (Perkin Elmer, Waltham, MA, USA). The percentage viability for each concentration in relation to the 0.0% concentration was calculated.

#### 2.2.5. Evaluation of the Expression of Toll-Like Receptors (TLR) TLR2 and TLR4 in the Cell Membrane by Flow Cytometry 

Tubes with 500,000 HaCaT cells, pretreated for 48 h with the sample (10 *C. acnes* thermo-inactivated bacteria per cell) and negative control (no treatment), were centrifuged at 800× *g*, and the supernatants were eliminated by aspiration. The pellets were washed twice with cytometry buffer (PBS, 5% BSA, 0.5% sodium azide) and resuspended in 100 µL of the same buffer and the anti-TLR2 and TLR4 antibodies according to manufacturer recommendations. The samples were incubated for 30 min, protected from light, and centrifugated and washed twice in the same way. Finally, cells were resuspended in 500 µL of the cytometry buffer and labeled with DAPI at a final concentration of 8 µg/mL. Samples were prepared in triplicates. The flow cytometry acquisition was performed using a Gallios Cytometer (Beckman Coulter, Brea, CA, USA).

#### 2.2.6. *Camellia sinensis* Anti-Inflammatory Activity Evaluation 

HaCaT cells were seeded in 6-well plates at 500,000 cells/well in 2 mL of media. After 24 h of incubation, the cells were not treated (negative control), treated with *C. acnes* (10 bacteria/cell) as positive control, or treated with *C. acnes* (10 bacteria/cell) + *C. sinensis* lysate (0.25% *w*/*w*). The cells were incubated at 37 °C, in 5% CO_2_, 95% air-humidified atmosphere for 6 h. Each condition was tested in biological triplicates, using cells from independent expansions.

After 6 h, cells were lysed and RNA was extracted with a RNeasy Mini Kit, according to suppliers’ instructions. The RNA obtained was quantified using the NanoDrop Lite (ThermoFisher, Waltham, MA, USA) and 1 µg of RNA was converted into cDNA, adjusting the volume to 16 µL with nuclease-free water, and then adding 4 µL of the iScript RT-PCR Supermix. The PxE Therman Cycler (ThermoFisher, Waltham, MA, USA) was used for the reverse transcription. After obtaining the cDNA, each sample was brought to the working concentration (10 ng/µL).

Finally, the expression of IL-6, IL-8, TNF-α, and CXCL1 was quantified by RT-PCR using LightCycler 480^®^ (Roche, Basel, Switzerland). For that purpose, 10 µL of LightCycler 480 Probes Master (1 U FastStart Taq DNA Polymerase, reaction buffer, 35 mM Tris (pH 8.4), 50 mM KCl, 0.25 mM dNTP mix, and 6.4 mM MgCl_2_), 5 µL of nuclease-free water, 1 µL of the corresponding TaqMan Gene Expression assays mix (Primer concentration 250 nM), and 4 µL of the corresponding sample were mixed. The thermal-cycling program was a preincubation cycle at 95 °C (10 min), followed by 40 cycles of 95 °C (10 s), 60 °C (30 s), 72 °C (1 s), and a final cooling cycle at 40 °C (30 s). Fluorescence signals were acquired at 60 °C for the quantification of the inflammatory mediators’ expression.

#### 2.2.7. NF-κB Nuclear Translocation Evaluation 

HaCaT cells were seeded in 6-well plates, in which a glass cover slip was placed at the bottom, at 500,000 cells/well in 2 mL of media. After an incubation period of 24 h, the cells were treated with the same treatment conditions and controls described in Section 2.2.6. 

After 1 h, the cells were washed with PBS and fixed (PFA 4% in PBS) for 10 min at room temperature (RT). Then, three quick PBS washes were performed, and the cells were permeabilized (0.2% Triton X-100 in PBS solution) for 15 min at RT. Thereafter, the cells were incubated in blocking solution (PBS, 3% BSA, 0.1% Tween-20) for 30 min at RT.

For staining, the samples were incubated with the antibody anti-NF-κB p65, conjugated with Alexa Fluor^®^ 488, diluted 1/50 in blocking solution, for 1 h at RT, and protected from light [32]. Then, five PBS washes of 5 min were performed. Posteriorly, the cells were incubated for 10 min in a 1 µg/mL DAPI solution.

The glass cover slips were taken from the bottom of the wells and placed on microscope slides with MOWIOL mounting medium overnight at RT. The samples were visualized and captured using the spectral confocal microscope Leica TCS-SPE (Leica Microsystems, Wetzlar, Germany). Before the final capture, a time-course experiment (carried out with the same conditions as described in this section) was performed to select the best time to conduct the final observation. The images were obtained with an epifluorescence microscope EVOS™ M5000 (ThermoFisher, Waltham, MA, USA).

The images were analyzed through a semi-quantitative evaluation of NF-κβ translocation, using the ImageJ software (Version 1.4.3, National Institutes of Health, Bethesda, MD, USA) for channel merging and for the evaluation in the cell nuclei of the green and blue fluorescence intensities. For the analysis, a line was drawn across each of the assessed nuclei to determine the blue and green fluorescence at each of the pixels forming the line. The total fluorescence of each color was evaluated.

#### 2.2.8. *C. acnes* Anti-Biofilm Activity of *Camellia sinensis* Callus Lysate 

Stock culture of *C. acnes* ATCC 6919 (isolated from human facial skin with acne lesion) was seeded in blood agar and incubated at 37 °C for 48 h. Later, bacteria were suspended on 10 mL RCM and the concentration was adjusted to a value between 1.5–5 × 10^8^ CFU/mL using the RCM. This suspension was used to develop the bacterial biofilm on 12-well flat-bottomed polystyrene plates. Borosilicate glass coupons were immersed in the bacterial suspension (control) and in the bacterial suspension with the *C. sinensis* callus lysate at two different concentrations: 2.5 and 5% *w*/*w*. The biofilm population density per coupon and planktonic cells in suspension were determined by quantitation of viable cells by serial dilutions and the plating method. A total of 100 µL of microbial suspension was plated onto blood agar and stored at 37 °C for 48 h in anaerobic conditions. The bacterial reduction was established as the difference between the viable cell counts obtained in the control coupons and those obtained in the coupons submerged in the test product suspensions after the contact time.

To visualize the biofilm, confocal laser scanning microscopy (CLSM) was used. For that purpose, each coupon was rinsed with ringer solution to eliminate the planktonic cells not adhered to the coupon and adding 10 µL of stains SYTO^®^ 9 and propidium iodide to each coupon (according to instructions from the Filmtracer LIVE/DEAD Biofilm Viability kit). 

#### 2.2.9. Antilipase Activity 

Lipase production was determined in biofilm cultures using the fluorogenic substrate 4-methylumbelliferyl (4-MU) oleate, according to method described by Coenye et al. [33]. Briefly, 200 µL of the substrate (0.2 mg/mL in DMSO) was mixed with 200 µL of *C. acnes* biofilm supernatant (recovered from coupon discs from Section 2.2.8) in black 96-well microtiter plates and incubated at 37 °C; the fluorescence (excitation wavelength: 355 nm and emission wavelength: 460 nm) was measured using a Varioskan LUX (ThermoFisher, Waltham, MA, USA) plate reader every 5 min for 45 min. The sterile RCM that was used to develop the *C. acnes* biofilm was added together with the substrate and considered as the medium control. The study was performed with three analytical replicates.

Lipase activity was expressed as relative fluorescence units (RFU) and calculated as the percentage reduction of lipase activity relative to *C. acnes* control (considered as 100% lipase activity) at the time of 45 min [34].

#### 2.2.10. Quantification of AI-2 

AI-2 production was detected in *C. acnes* during the initial stage of the biofilm formation process (Section 2.2.8), after 24 h of incubation at 37 °C in TS Broth medium. Detection of AI-2 was carried out according to the method described by Taga et al. [35] employing *V. harveyi* strain BB170, a bioluminescent strain that produces light in response to AI-2. The culture medium was used as a negative control. The AI-2-producing *V. harveyi* BB152 strain was used as a positive control. Thus, 180 µL of the *V. harveyi* strain BB170 suspension was mixed with 20 µL of the *C. acnes* initial biofilm medium suspension (recovered at 24 h) in white 96-well microtiter plates and incubated at 37 °C; the light produced (in response to AI-2) was measured in each sample at a wavelength of 490 nm, every 10 min for 4 h. The study was performed with three analytical replicates.

The luminescence RLU values at time 210 min (maximum value of AI-2 in *C. acnes* control) were used to calculate the percentage decrease in AI-2 production. 

#### 2.2.11. Statistical Analysis of the Results 

For the statistical analysis of the results, the homogeneity of variance was confirmed by the Levene test, and the normality was confirmed by the Anderson–Darling test. Unpaired t-test and one-way analysis of variance (ANOVA) combined with Bonferroni’s test were conducted to assess differences between analysed groups with respect to *C. acnes* control group without treatment. P values less than 0.05 were designated as significant differences. Statistical analyses were conducted by GraphPad Prism 9 software (GraphPad Software v7, Inc., San Diego, CA, USA).

## 3. Results

### 3.1. Chemical Characterization of the Camellia sinensis Callus Extract

The total terpenoid content, expressed as a function of β-caryophyllene, was shown to be 8.08 g/L (CV = 18.04%).

**Figure 1:**
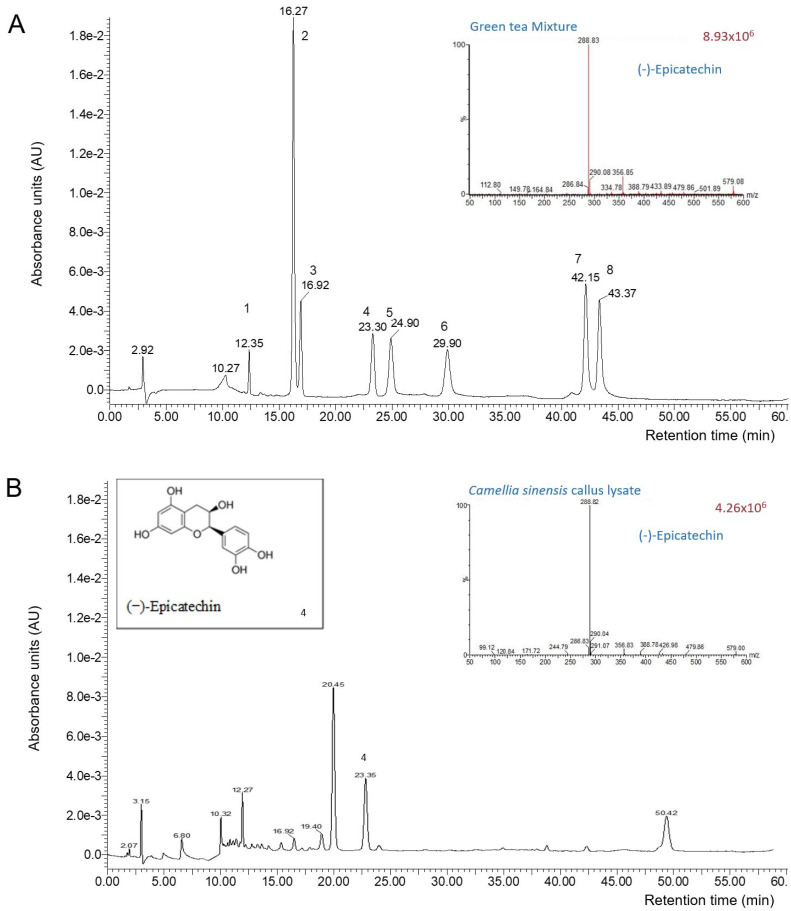
Panel (**A**): green tea mixture (1: (−)-gallocatechin; 2: caffeine; 3: (+)-catechin; 4: (−)-epicatechin; 5: (−)-epigallocatechin-3-galate; 6: (−)-gallocatechin galate; 7: (−)-catechin 3-gallate; 8: epicatechin 3-gallate). Panel (**B**): representative chromatogram of *Camellia sinensis* callus cell lysate (1: (−)-epicatechin).

The most characterized compounds in green tea extract are classically the polyphenols, especially the catechins, because of their multiple biological properties (antioxidant, antimicrobial, anti-inflammatory, antiviral, and anti-cancer, among others) [36,37]. A mixture of green tea catechins was used to identify the peaks of each compound (Figure 1A). Epicatechin (EC, retention time 23.3 min; *m*/*z* ratio 288.9) represented 94% of the catechins found in the lysate (in terms of chromatographic purity, Figure 1B), which corresponds to 179.40 µg/mL (CV = 5.1%). Considering the fact that the *C. sinensis* lysate is a freeze-dried product, this concentration is equivalent to 2.11 mg EC/g of freeze-dried lysate.

### 3.2. In Vitro Anti-Inflammatory Effect of Camellia sinensis Callus Cells Extract

Before assessing the anti-inflammatory efficacy of the callus lysate, two experiments were performed to set up the experimental conditions for the efficacy study. These assessed the cytotoxicity of the lysate (to select the appropriate concentration) and the expression of toll-like receptor (TLR) 2 and 4 (to validate the inflammatory stimulus).

**Figure 2:**
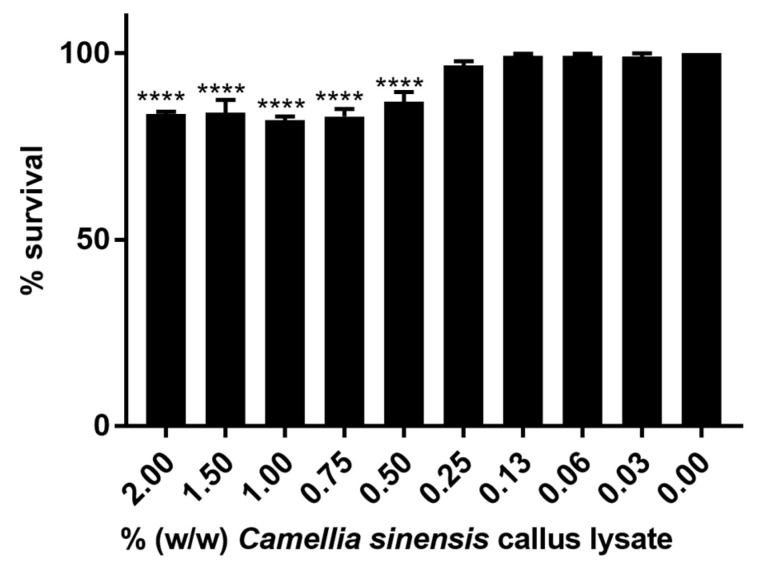
HaCaT cell viability results after treatment with different concentrations of *Camellia sinensis* callus lysate. Statistical differences: **** (*p* < 0.0001).

Figure 2 shows the result of the cytotoxicity assessment of the callus lysate using the HaCaT cell line, where a clear dose–response can be observed. A concentration of 0.25% *w*/*w* was the highest concentration that did not show statistically significant differences with regard to the control (no treatment). Therefore, this concentration was selected for the following studies.

The bacteria *C. acnes* American Type Culture Collection (ATCC) 6919 was selected as the inflammatory stimulus and is considered the most representative model of inflammatory response for acne disease. This bacterial strain was originally obtained from patient acne lesions. Thereafter, it was determined that this ATCC strain corresponds to phylotype IA_1_ [29,30,31]_,_ which is one of the most severe strains in acne patients [38]. 

**Figure 3:**
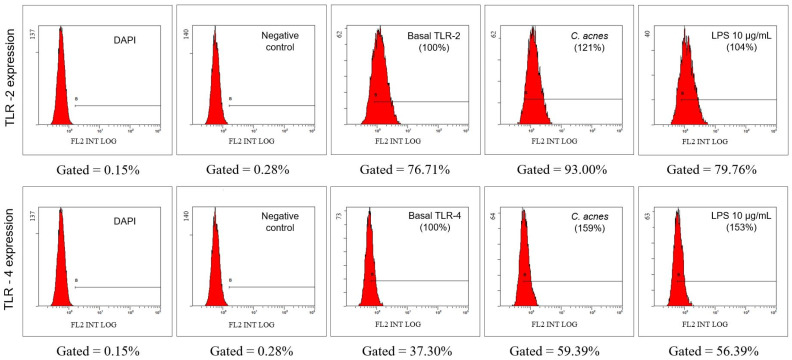
TLR- 2 and TLR-4 expression in HaCaT cells from the flow cytometry of control cells (DAPI and no treatment), basal value, and stimulated with thermo-inactivated *Cutibacterium acnes* (10 bacteria/cell) or with lipopolysaccharide (10 µg/mL).

The inflammatory response produced in acne is mediated through the detection of the *C. acnes*-pathogen-associated molecular patterns (PAMP), mainly peptidoglycan and lipoteichoic acid, by TLR2 and TLR4 [39,40]. Lipopolysaccharide (LPS) is a characteristic compound of Gram-negative bacteria, and it is a commonly used inflammatory stimuli, but its effect is mediated by the stimulation of TLR4 only and has shown a limited capacity to stimulate TLR2 [41]. On the other hand, TLR2 has a major role in the recognition of Gram-positive bacteria, such as *C. acnes* [42]. Figure 3 shows the flow cytometry results of both TLR2 and TLR4 receptors. In addition, the relative receptor expression compared to control was calculated. 

As can be seen, the thermo-inactivated bacillus (10 bacteria/cell) induced the expression of both TLR2 and TLR4, so it was used as the inflammatory stimulation for the following experiments. 

**Figure 4:**
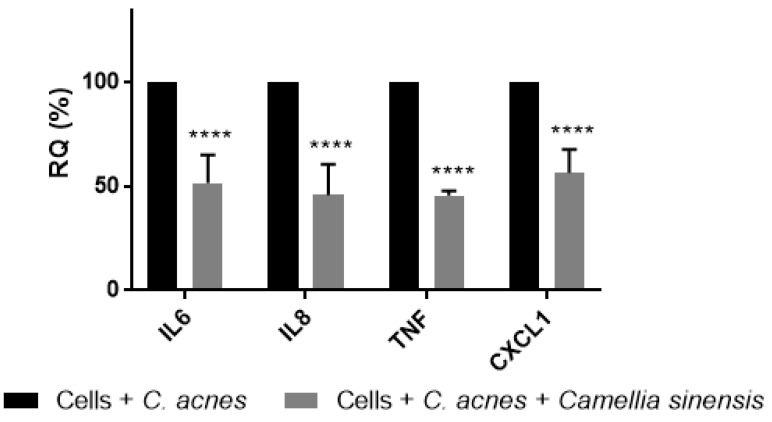
Cytokine and chemokine reduction of HaCaT cells stimulated with *Cutibacterium acnes* after treatment with *Camellia sinensis* callus lysate at 0.25% *w*/*w*. RQ: relative quantification. Statistical differences: **** (*p* < 0.0001).

Thermo-inactivated *C. acnes* in contact with HaCaT cells induced the production of the tested interleukins IL-6 (0.5-fold increase), IL-8 (2.4-fold increase), TNF-α (2.3-fold increase), and CXCL1 (1.9-fold increase). Otherwise, co-incubation with *C. sinensis* callus cell lysate at 0.25% (corresponding to 20.20 µg/mL of terpenes and 0.45 µg/mL of EC) significantly reduced their expression (Figure 4). IL-6 exhibited an expression after treatment of 51.33 ± 13.65%, IL-8 45.67 ± 14.57%, TNF-α 45.67 ± 2.08%, and CXCL1 56.33 ± 11.02%, which correspond to a reduction of 48.67, 54.33, 54.33, and 43.67%, respectively. 

**Figure 5:**
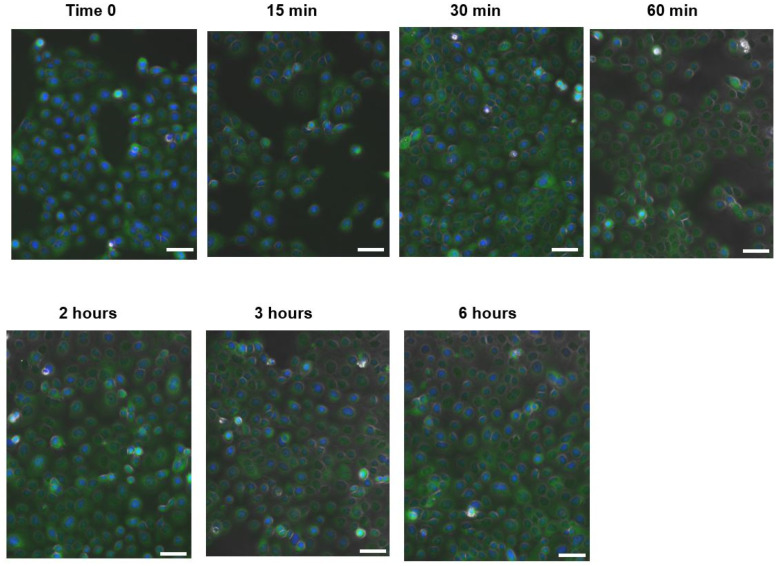
Time-course experiment using HaCaT cells stimulated with *Cutibacterium acnes*. Blue color corresponds to the nucleus (DAPI staining). Green color corresponds to NK-kB (Alexa Fluor 488). Bar represents 100 µm.

As previously described, the induction of these inflammatory mediators is caused by transcription factor NF-κB. To evaluate its translocation to the nucleus, a confocal microscopy experiment was carried out. Prior time-course experiments were performed (Figure 5) to select the most appropriate time after cell stimulation with *C. acnes*. An intensity peak was observed at 1 h after stimulation (the higher green intensity on the cell nucleus).

**Figure 6:**
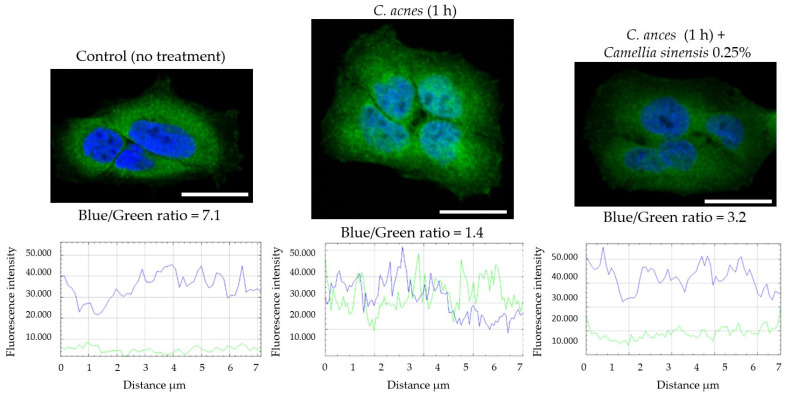
Confocal microscopy of HaCaT cells stimulated with *Cutibacterium acnes* after 1 h and treated with *Camellia sinensis* callus lysate. Blue color corresponds to the nucleus (DAPI staining). Green color corresponds to NF-κB (Alexa Fluor 488). Below each image, the intensity plots and blue/green ratio are reported. Bar represents 20 µm.

Once the time point was selected, the final experiment focused on NF-κB translocation to the nucleus (Figure 6) was conducted. As can be seen, control cell growth exhibited a blue nucleus due to 4′,6-diamidino-2-fenilindol (DAPI) staining, with no green signal corresponding to NF-κB. Cells stimulated with *C. acnes* exhibited a marked green fluorescence due to the Alexa Fluor^®^ 488 fluorochrome. Finally, cells treated with *C. sinensis* callus lysate exhibited a less intense green color, showing the reduction in NF-κB activation correlating with the lower expression of cytokines and chemokines in the previous experiment after the treatment with the same lysate at the same concentration. A semi-quantification of the fluorescent nucleus blue/green intensity ratio was performed with the ImageJ software in six different cells per treatment. The higher the ratio value, the less the transcription factor translocated to the nucleus and the lower the levels of inflammation. The intensity plot and fluorescent blue/green ratio are shown in Figure 6. 

### 3.3. In Vitro Anti-Biofilm Activity 

To evaluate the potential antibiofilm activity (adhesion, growth, and viability) of *C. sinensis* callus lysate, an in vitro study with mature biofilms was carried out. An initial inoculum of *C. acnes* ATCC 6919 corresponding to 1.7 × 10^8^ CFU/mL was grown in 12-well polystyrene plates. To obtain the mature biofilms, borosilicate glass coupons were immersed in a Petri dish with the bacterial suspension for 5 days, and the herbal lysate activity was then evaluated. 

**Figure 7:**
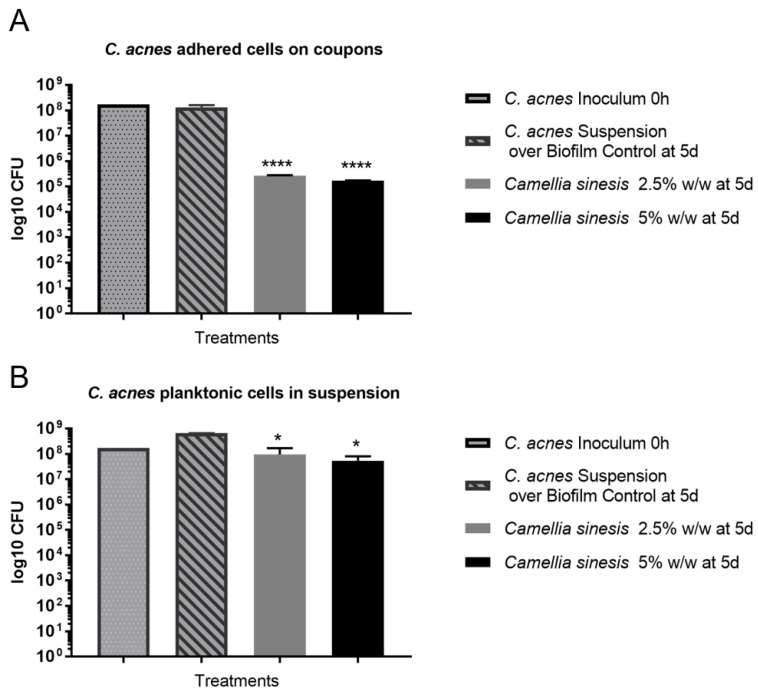
Panel (**A**): *Cutibacterium acnes*-adhered cell (biofilm) population (log10 CFU) of the control (no treatment) over the mature biofilm after the treatment with 2.5 and 5% *w*/*w Camellia sinensis* callus lysate. Panel (**B**): *Cutibacterium acnes* planktonic cell population (log10 CFU) of the control (no treatment) over the mature biofilm and after the treatment with 2.5 and 5% *w*/*w Camellia sinensis* callus lysate. Statistical differences compared with control: * (*p* < 0.05); **** (*p* < 0.0001).

Figure 7A shows the colony-forming units (CFU), expressed as log_10_, of the negative control (no treatment), and the herbal lysate at two different concentrations (2.5 and 5% *w*/*w*, corresponding to 202.00 and 404.00 µg/mL of terpenes and 4.49 and 8.97 µg/mL of EC, respectively). Figure 7B illustrates the viability of planktonic bacteria in the supernatant. The reduction in CFU/mL on the biofilms was 2.69 ± 0.11 and 2.89 ± 0.10 log_10_-units for the concentrations of 2.5 and 5% *w*/*w*, respectively. The logarithmic reductions on planktonic cells were 0.91 ± 0.31 and 1.12 ± 0.19, respectively. In both cases, the CFU reduction was statistically different. 

**Figure 8:**
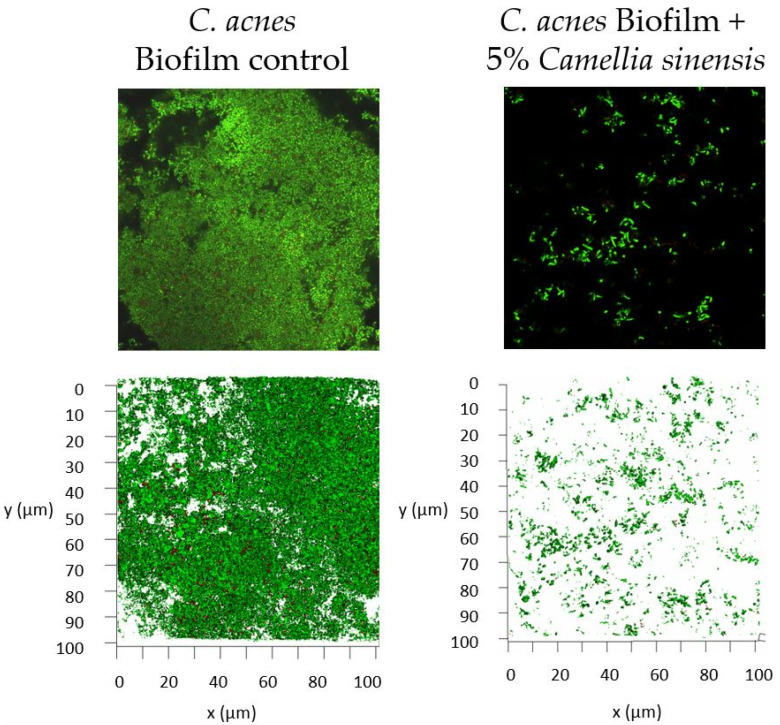
Confocal microscopy images with 63X objective of the control (no treatment) mature biofilm of *Cutibacterium acnes* and after treatment with 5% *w*/*w Camellia sinensis* callus lysate. Upper images correspond to direct visualization. Lower panels correspond to a 3D reconstruction with 63X objective.

Figure 8 shows the biofilm image after the treatment with the callus lysate at 5%, as compared to control (no treatment). The SYTO^®^ 9 stain stains all bacteria nucleic acid with green fluorescence showing those with intact membranes and those with damaged membranes. In contrast, propidium iodide penetrates only bacteria with damaged membranes, causing a reduction in the SYTO^®^ 9 stain fluorescence when both dyes are present. Thus, bacteria with intact cell membranes appear fluorescent green, whereas bacteria with damaged membranes appear fluorescent red. As can be seen, a significant reduction in the biofilm density was observed after the treatment. These images confirm the results obtained in the colony-counting experiment. 

### 3.4. In Vitro Anti-Lipase Activity

**Figure 9:**
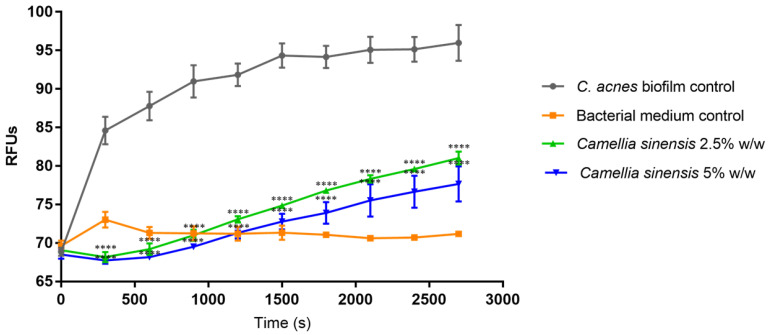
*Cutibacterium acnes* lipase activity expressed as relative fluorescence units (RFU) measured at different times over 45 min ofthe control (untreated) mature *Cutibacterium acnes* biofilm (black line) and after treatment with 2.5% *w*/*w* (blue line) or 5% *w*/*w* (green line) *Camellia sinensis* callus lysate. Bacterial medium used to develop *Cutibacterium acnes* biofilm is denoted by the red line. Statistical differences: **** (*p* < 0.0001).

A fluorescent dye was incubated with the *C. acnes* biofilm supernatant and the fluorescence of the hydrolyzed metabolite was quantified at several times. The results are reported in Figure 9. Considering the last time point evaluated, the lipase levels of the positive control (*C. acnes* biofilm with no treatment), and the basal fluorescence of the medium, the herbal lysate at 2.5% *w*/*w* inhibited the lipase activity by 60.05 ± 5.80% and the concentration of 5% *w*/*w* reduced the lipase activity by up to 73.14 ± 12.12%. Both reductions were statistically different compared to the *C. acnes* control (*p* < 0.0001). 

Therefore, these results, together with the antibiofilm activity, corroborate the hypothesis concerning the reduction in virulence factors exerted by *C. sinensis* callus lysate in *C. acnes* cultures. 

### 3.5. Quantification of Autoinducer 2 (AI-2) 

**Figure 10:**
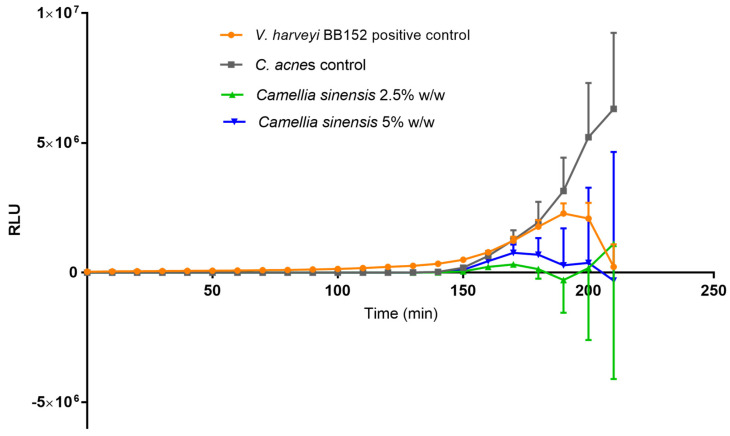
Production of AI−2 expressed as relative luminescence units (RLU) measured at different times over 210 min in the control (untreated) mature *Cutibacterium acnes* biofilm (gray line) and after treatment with 2.5% *w*/*w* (green line) and 5% *w*/*w* (blue line) *Camellia sinensis* callus lysate. The AI−2−producing *Vibrio harveyi* BB152 positive control is denoted by the orange line.

Finally, the production of AI-2 in *C. acnes* biofilms was quantified. For that purpose, *V. harveyi* strain BB170 was used as the detector of the AI-2 content secreted by *C. acnes* on the medium. In the presence of AI-2, *V. harveyi* acts as a biosensor and produces luminescence. Firstly, a kinetics of AI-2 production was carried out (Figure 10) with two controls, *C. acnes* biofilm with no treatment and *V. harveyi* BB152, and two different concentrations of *C. sinensis* callus lysate (2.5 and 5.0%). As can be seen, the *C. acnes* AI-2 production increased considerably, which suggests that significant communication between bacteria occurred at the beginning of biofilm formation. The bacteria *V. harveyi* BB152 was used as the positive control for AI-2 production. The AI-2 production in this bacterium had a different kinetics profile than that of *C. acnes*; the positive control reached the maximum and the test bacteria biofilm continued growing. Finally, the callus lysate, at the end of the experiment, was able to reduce AI-2 production by 102.44 ± 91.50% at the lower concentration, and 141.51 ± 118.80% at the higher concentration. 

## 4. Discussion

Terpenes are compounds that are often related to the fragrance and taste of plants, but they are also involved in other plant functions, such as signaling pathways and defence against insects and bacteria. Moreover, they are also used as a result of their anti-inflammatory and anti-cancer properties in the human body [22]. Many terpenes are synthesized in consequence to external or internal plant stressors or as a signaling function; then, its production depends a lot on the external conditions. The technological approach to produce the lysate obtained in this article uses different elicitors to stimulate the production of second metabolites; then, the terpene production is highly dependent on the used elicitors. Previous studies [43] showed that the level of terpene content in *C. sinensis* flowers or leaves are on the range of ppm (ug/kg of dry matter). Other authors evaluated the number of non-volatile terpenes of *C. sinensis* but did not quantify their content [44] or performed a comparative study within the volatile compound content, but not an absolute quantification of terpene content [45]. Since the compounds with biological properties most described and studied were green tea polyphenols, the terpene study has been classically addressed for studies of its sensory properties.

Contrary to other extracts, in this case, EC is the major component. The main components of traditional solvent-extracted green tea are usually epigallocatechin, epigallocatechin gallate, or epicatechin gallate. The callus lysate used in this research was elicited with different stressors to modulate secondary metabolite production. This could be the reason for the different catechin profile as compared with those observed using traditional extraction methods.

The thermo-inactivated *C. acnes* induced the expression of both TLR2 and TLR4. Both receptors are expressed on several immune-related cell types, including macrophages, neutrophils, dendritic cells, and nonimmune-related cells (fibroblasts and keratinocytes). Once activated, the signaling cascade leads to NF-κB activation and the expression of proinflammatory cytokines and chemokines through keratinocytes, which increase the adaptive immunity [46]. IL-6 and TNF-α induce the activation of T helper 17 leukocytes (Th17). IL-8 modulates the expression of the adhesion molecules involved in the recruitment of neutrophils [47] and chemokines, such as CXCL1, which are also expressed by keratinocytes after an inflammatory response to induce chemotaxis [48,49].

The lysate was able to reduce the inflammation mediated by the interaction of *C. acnes* ATCC6919 with TLR 2 and TLR4 in keratinocytes. A mixture of 0.25% *w*/*w* of the herbal lysate (corresponding to 20.20 µg/mL of terpenes and 0.45 µg/mL of EC) significatively reduced the expression of IL-6, IL-8, TNF-α, and CXCL1, and reduced NF-κB translocation to the nucleus, demonstrating its anti-inflammatory effects. Other authors [18] used a similar in vitro model to evaluate the anti-inflammatory effect of *C. sinensis* traditional extracts, but with different cell lines and using LPS as the inflammatory stimuli; for this reason, the results are difficult to compare. In different studies, other herbal extracts (*Buddleja davidii* aqueous extract [50], *Helichrysum odoratissimum* [15], a nd *Rosa davurica* [51]) were tested in HaCaT cells stimulated by *C. acnes*, but the experimental conditions were slightly different, and the results are not directly comparable. In fact, no in vitro anti-inflammatory study focused on HaCaT keratinocyte cells using *C. acnes* bacteria as the inflammatory stimuli to test *C. sinensis* callus lysate was found in the literature.

A concentration of between 2.5 and 5% *w*/*w* of the lysate (corresponding to 202.00 and 404.00 µg/mL of terpenes and 4.49 and 8.97 µg/mL of EC, respectively) significatively reduced *C. acnes* biofilm formation in vitro. The product under study did not exhibit a high antibacterial activity when the cells were in suspension (planktonic bacteria), but the CFU reduction was higher when the biofilm was formed. It was generally assumed that the antibacterial activity of green tea extract is related with a direct bactericidal mechanism. There are many publications that determine the minimum inhibitory concentration (MIC) or inhibitory area using the agar method. These results demonstrate the high potency of the traditional extract to inhibit the growth of several bacteria [52,53,54]. The lower log-reduction observed in the bacterial suspension (as compared to the biofilm) may indicate that the *C. sinensis* callus lysate limited bacteria–bacteria interactions, causing a reduction in the adhesiveness and/or limiting or blocking the virulence factors. This is an important fact as *C. acnes* is part of the skin microbiota and has a physiological role in skin homeostasis [55]. There is increasing evidence to support the hypothesis that acne is more related with the loss of microbiota diversity than with the presence of a specific micro-organisms [38,56]. Therefore, the therapeutic approach to treating acne patients should be to limit the proliferation of *C. acnes* to maintain microbiota balance. The results obtained with the *C. sinensis* callus lysate imply this. Instead of obtaining a high bactericidal effect, the lysate seems to limit the proliferation of *C. acnes* (low-viability reduction of planktonic cells) and restrict the induction of virulence factors (reduction of biofilm [57]). This mechanism could be related to the decrease in cell-to-cell communication or the quorum-sensing bacterial mechanism [58]. 

Lipase activity is another well-known virulence factor of *C. acnes*, which contributes to the development of acne lesions. The *C. acnes* lipase hydrolyzes the skin triglycerides into the corresponding free fatty acids. These compounds increase inflammation and worsen the pathology [59]. The approach of reducing the lipase activity of *C. acnes* as a treatment option for acne has already been addressed by other authors using various compounds. Abozeid et al. [16] used ketoconazole and obtained a reduction in the lipase activity of *C. acnes* of between 60–80% with drug concentrations between 8–16 µg/mL; Nakase et al. [60] described a reduction in lipase activity trough the inhibition of the expression of extracellular lipase gene gehA; and Wunnoo et al. [61] achieved a significant reduction of lipase activity using a Rhodomyrtone treatment in a concentration of 8 mg/mL, but the antilipase effect of *C. sinensis* derivatives had not been described until now. The results showed that the herbal lysate exerted a statistically significant reduction in this virulence factor in *C. acnes* cultures at both tested concentrations. In addition, this new *C. sinensis* callus lysate also reduced the AI-2 quoro-hormone levels, which confirmed the quorum-quenching activity of the *C. sinensis* callus lysate, rather than a bacteriolytic activity. The proposed mechanism of action would improve the treatment of acne, as our understanding of the dysbiosis among the different bacterial populations has increased in importance as the basis of this pathology. The aim of the treatment should not be the eradication of *C. acnes*, because it belongs to the commensal microbiome and has a physiological role in skin homeostasis. On the contrary, the ideal therapy should balance the different bacterial populations in a normal status and reduce the virulence factors derived from the overgrowth of specific bacteria. Further clinical studies are needed to achieve more conclusive and reliable results regarding the use of this *C. sinensis* callus lysate for the management of acne vulgaris, taking into account the remarkable side effects exerted by conventional therapies.

## Data Availability

Data are available upon request due to intellectual property.

## Supplementary Materials

The following supporting information can be downloaded at: https://www.mdpi.com/article/10.3390/cimb45050255/s1. *Camellia sinensis* anti-inflammatory activity evaluation, detailed information of primers and probes.
