# Supplementary material for: Anti-Inflammatory and Anti-Quorum Sensing Effect of Camellia sinensis Callus Lysate for Treatment of Acne
Source: Curr Issues Mol Biol. 2023 May 4;45(5):3997–4016. doi: 10.3390/cimb45050255 (PMC10217533; doi:10.3390/cimb45050255)
Supplement: Supplementary file 1 [file cimb-45-00255-s001.zip › cimb-2249839-supplementary.pdf]

***Camellia sinensis* anti-inflammatory activity evaluation, detailed information of primers and probes.**

Primers used for the determination of cytokines and chemokines were commercial. Complete description of used primers and probes::

- Hs00174103_m1,
  - Gene Symbol:CXCL8
  - Gene Name: C-X-C motif chemokine ligand 8Gene
  - Aliases:GCP-1, GCP1, IL8, LECT, LUCT, LYNAP, MDNCF, MONAP, NAF, NAP-1, NAP1
  - Chromosome Location:Chr.4: 73740506 - 73743716 on Build GRCh38
  - Species:Human
  - RefSeq NM_000584.3 NP_000575.1
- Hs00174128_m1,
  - Gene Symbol: TNFGene Name:tumor necrosis factorGene
  - Aliases: DIF, TNF-alpha, TNFA, TNFSF2, TNLG1F
  - Chromosome Location:Chr.6: 31575567 - 31578336 on Build GRCh38
  - Species: Human
  - RefSeq NM_000594.3 NP_000585.2
- Hs00174131_m1,
  - Gene Symbol:IL6
  - Gene Name :interleukin 6
  - Gene Aliases:BSF-2, BSF2, CDF, HGF, HSF, IFN-beta-2, IFNB2, IL-6
  - Chromosome Location:Chr.7: 22725889 - 22732002 on Build GRCh38
  - Species: Human
  - RefSeq NM_000600.4 NP_000591.1
- Hs00236937_m1,
  - Gene Symbol:CXCL1
  - Gene Name: C-X-C motif chemokine ligand 1
  - Gene Aliases: FSP, GRO1, GROa, MGSA, MGSA-a, NAP-3, SCYB1
  - Chromosome Location:Chr.4: 73869392 - 73871302 on Build GRCh38
  - Species: Human
  - RefSeq NM_001511.3 NP_001502.1
- Hs02786624_g1, housekeeping gene for calculation of relative quantification

- Gene Symbol: GAPDHGene
- Name:glyceraldehyde-3-phosphate dehydrogenase
- Gene Aliases:G3PD, GAPD, HEL-S-162eP
- Chromosome Location:Chr.12: 6534405 - 6538375 on Build GRCh38
- Species: Human
- RefSeq NM_001256799.2, NP_001243728.1

The concentrations of primers in the TaqMan assays were: 18 μM for Forward Primer, 18 μM for Reverse Primer and 5 μM for the fluorescent Probe. Primers are kept at -20ºC.
